# Draft Genome Sequence of *Streptomyces vitaminophilus* ATCC 31673, a Producer of Pyrrolomycin Antibiotics, Some of Which Contain a Nitro Group

**DOI:** 10.1128/genomeA.01582-15

**Published:** 2016-01-21

**Authors:** Kristina M. Mahan, Dawn M. Klingeman, Robert L. Hettich, Ronald J. Parry, David E. Graham

**Affiliations:** a Biosciences Division, Oak Ridge National Laboratory, Oak Ridge, Tennessee, USA; b Chemical Sciences Division, Oak Ridge National Laboratory, Oak Ridge, Tennessee, USA; c Department of Chemistry and Department of Biochemistry and Cell Biology, Rice University, Houston, Texas, USA

## Abstract

*Streptomyces vitaminophilus* produces pyrrolomycins, which are halogenated polyketide antibiotics. Some of the pyrrolomycins contain a rare nitro group located on the pyrrole ring. The 6.5-Mbp genome encodes 5,941 predicted protein-coding sequences in 39 contigs with a 71.9% G+C content.

## GENOME ANNOUNCEMENT

*Streptomyces vitaminophilus* (formerly *Actinosporangium vitaminophilum*) is one of several species of *Streptomyces* that are known to produce a family of halogenated antibiotics called the pyrrolomycins (1–6). These compounds exhibit potent antibiotic activity against Gram-positive bacteria, and they inhibit substance P-induced release of myeloperoxidase from human polymorphonuclear leukocytes (7). The most unusual structural feature of the pyrrolomycins is the presence of a nitro group located on the pyrrole ring in some of the antibiotics (5, 8). Natural products that contain nitro groups are uncommon, and relatively little is known about the biochemistry of nitro group formation (9). Although the pyrrolomycin biosynthetic gene clusters have been cloned from *Streptomyces vitaminophilus* and *Streptomyces* sp. UC 11065, sequence analysis of the gene clusters did not reveal the mechanism for nitro group formation in these antibiotics (10).

An Illumina TruSeq paired-end library was prepared with an insert size of approximately 584 bp and sequenced using an Illumina MiSeq instrument (390-fold coverage). Pacific Biosciences single-molecule, real-time DNA sequencing was performed by the University of Maryland School of Medicine Genomics Resource Center using P4-C2 chemistry (44-fold coverage). The genome sequence was assembled from trimmed and corrected Illumina data and preassembled Pacific Biosciences reads using the SPAdes genome assembler version 3.6.1 (11) and PBJelly software version 18.5.24 (12). The assembled genome sequence was polished using merged paired-end Illumina data with Bowtie2 version 2.2.5 (13) and Pilon software version 1.13 (14).

The draft genome sequence of *Streptomyces vitaminophilus* ATCC 31673 included 6,549,812 bp with a G+C content of 71.9%. The assembled genome comprises 39 contigs with an *N*_50_ of 249,406 bp and an *L*_50_ of 8 contigs. Coding DNA sequences (CDSs) were identified and annotated by the NCBI Prokaryotic Genome Annotation Pipeline. The genome was predicted to contain 5,941 CDSs, 56 tRNAs, two 16S rRNAs, two 23S rRNAs, and four 5S rRNAs.

A 227-kbp contig includes the entire 56-kbp pyrrolomycin biosynthetic gene cluster previously deposited to GenBank (accession number EF140901.1) (10). There are no recognizable homologs of nitric oxide synthases or previously characterized *N*-oxygenases; however, the genome encodes 15 cytochrome P450 homologs, two assimilatory nitrate reductases, and one nitrite reductase, which could be involved in bionitration.

Previous studies have shown that nitric oxide synthase inhibitors do not adversely affect nitrated pyrrolomycin biosynthesis in *Streptomyces fumanus*, suggesting a completely novel bionitration reaction (15). AntiSMASH version 3.0 (16) predicted 27 biosynthetic gene clusters, including genes for nonribosomal peptide synthases, type I, II, and III polyketide synthases, pyrrolomycins, siderophores, terpenes, lantipeptides, and even a lassopeptide. *Streptomyces vitaminophilus* ATCC 31673 produces the pyrrolomycins A–D, of which pyrrolomycins A and B contain a nitro group. Few enzymes involved in nitro group formation have been identified. The availability of the *Streptomyces vitaminophilus* genome and pyrrolomycin biosynthetic gene cluster sequences will facilitate the future identification of bionitration enzymes and add to the knowledge about nitro group formation.

### Nucleotide sequence accession numbers.

This whole-genome shotgun project has been deposited at DDBJ/EMBL/GenBank under the accession number LLZU00000000. The version described in this paper is the first version, LLZU01000000.
